# Data on mathematics teacher educators’ proficiency and willingness to use technology: A structural equation modelling analysis

**DOI:** 10.1016/j.dib.2024.110307

**Published:** 2024-03-11

**Authors:** Angel Mukuka

**Affiliations:** Department of Mathematics, Science and Technology Education, Walter Sisulu University, Mthatha, South Africa

**Keywords:** Mathematics teacher educators, Perceived ease of use, Perceived usefulness, Proficiency and willingness, Structural equation modelling, Technology integration

## Abstract

The role of Mathematics Teacher Educators (MTEs) in preparing future teachers to effectively integrate technology into their mathematics instruction is of paramount importance yet remains an underexplored domain. Technology has the potential to enhance the development of 21st-century skills, such as problem-solving and critical thinking, which are essential for students in the era of the fourth industrial revolution. However, the rapid evolution of technology and the emergence of new trends like data analytics, the Internet of Things, machine learning, cloud computing, and artificial intelligence present new challenges in the realm of mathematics teaching and learning. Consequently, MTEs need to equip prospective teachers with the knowledge and skills to harness technology in innovative ways within their future mathematics classrooms. This paper presents and describes data from a survey of 104 MTEs in Zambia. The study focuses on MTEs' proficiency, perceived usefulness, perceived ease of use, and willingness to incorporate technology in their classrooms. This data-driven article aims to unveil patterns and trends within the dataset, with the objective of offering insights rather than drawing definitive conclusions. The article also highlights the data collection process and outlines the procedure for assessing the measurement model of the hypothesised relationships among variables through structural equation modelling analysis. The data described in this article not only sheds light on the current landscape but also serves as a valuable resource for mathematics teacher training institutions and other stakeholders seeking to understand the requisites for MTEs to foster technological skills among prospective teachers of mathematics.

Specifications Table

**Table:** 

Subject	Mathematics Education
Specific subject area	Technology Integration in Mathematics Teacher Education
Data format	Raw Analyzed
Type of data	.csv file (raw numeric dataset for ordinal, categorical and scale variables) .csv file (raw textual dataset for all ordinal and categorical variables) .sav file (raw SPSS dataset) Tables (analysed data within the article for reliability and validity checks) Figure (analysed data within the article and the supplementary files on measurement model assessment of the structural relationships among key variables)
Data collection	These data were collected from Zambian Mathematics Teacher Educators (MTEs) in both private and public institutions using a descriptive cross-sectional research design. The focus was on lecturers who train secondary school mathematics teachers. A semi-structured questionnaire, created via Google Forms, was distributed online. Participants were selected through snowball and convenience sampling methods due to the voluntary nature of the study and the aim to recruit as many participants as possible. The author reached out to easily accessible MTEs and some Ministry of Education officials for wider distribution. Data collection occurred from mid-June to early August 2023.
Data source location	Data were gathered from a diverse group of 104 MTEs. These MTEs are affiliated with a wide range of institutions across Zambia, including 12 universities and 16 colleges of education. Notably, these institutions are geographically dispersed, with representation in all 10 provinces of Zambia. This broad coverage ensures a well-rounded perspective from MTEs across the entire country.
Data accessibility	The data described in this article are openly available at: Repository name: Mendeley Data Data identification number: Doi. 10.17632/3x8gs6nkk8.1 Direct URL to data: https://data.mendeley.com/datasets/3x8gs6nkk8/1

## 1 Value of the Data

• The value of the dataset described in this article lies in its ability to reveal the current state and needs of MTEs in Zambia regarding technology integration in mathematics teacher education.
• The dataset provides a basis for future studies and initiatives that aim to improve the technological competencies and perspectives of MTEs in Zambia.
• The data benefits MTEs seeking to boost their technological proficiency and enthusiasm, as well as mathematics teacher training institutions aiming to develop effective curricula and programs for MTEs’ professional development.
• The data described in this paper could potentially support policymakers in making informed decisions and resource allocation to foster technology integration in mathematics teacher education.
• The dataset provides insights into how other researchers can replicate the study in other countries or regions to compare the results and identify commonalities and differences.
• Future researchers in this domain may also extend the study by adding more variables or using different methods to explore the relationships among MTEs’ proficiency, perceived usefulness, perceived ease of use and willingness to use technology.

## 2 Background

In an era where technology is increasingly intertwined with everyday life, the integration of technology into mathematics education is a pressing issue, especially in low-resource settings like Zambia. In light of this, a dataset was compiled to explore the under-researched domain of MTEs’ proficiency and willingness to integrate technology into their instruction. This exploration is essential as it provides a comprehensive insight into the pivotal role that MTEs play in enhancing the technological competence of preservice mathematics teachers. As the primary facilitators of mathematics teacher education, MTEs are instrumental in shaping the technological proficiency of future teachers. Their understanding of, and ability to effectively integrate, technology into their teaching practices directly influences the technological readiness of their students. Therefore, this dataset [1] serves as a catalyst for targeted interventions and strategies, which in turn, has the potential to elevate the quality of mathematics education overall.

**Fig. 1:**
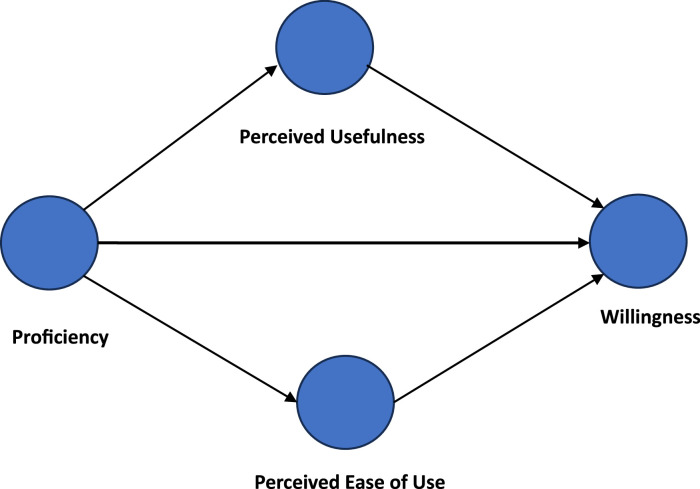
Hypothesised structural relationships among study variables.

The theoretical underpinning for this study is grounded in the Technology Acceptance Model (TAM) and Technological Pedagogical Content Knowledge (TPACK). These models provide a framework for understanding the factors influencing MTEs’ acceptance and use of technology. According to the TAM, an individual's willingness to adopt technology is primarily determined by their perception of its usefulness and ease of use [2]. In the context of this paper, the proficiency of MTEs with digital technology, evaluated using the TPACK model [3], is considered an external variable. It is hypothesized that this proficiency could influence an individual's perceived usefulness and ease of use of technology, ultimately affecting their willingness to use it, as depicted in Fig. 1.

The collected data focuses on MTEs’ proficiency, perceived usefulness, perceived ease of use, and willingness to incorporate technology in their classrooms. This paper outlines the process of data collection and the methodology employed for evaluating the measurement model. This model hypothesizes relationships among the variables and is assessed through a partial least squares structural equation modelling (PLS-SEM) analysis. The assessment adheres to the criteria set forth by Ringle et al. [4] in their perspective paper on the application of PLS-SEM in data articles. This adherence ensures that the model aligns meaningfully and consistently with the collected data. Key aspects such as the scale levels of data, the representativeness of the sample, and the outcomes of the reflective model assessment are thoroughly highlighted. Supporting evidence for the fit of the structural model is also discernible through the reported coefficients of determination. However, this paper does not delve into the percentage of missing values, the missing data option utilized, or the comprehensive results of the structural model. The omission of the missing data aspect is justified as the prevalence of missing values was not significant enough to impact the validity and reliability of the results.

## 3 Data Description

Three different versions of the same dataset are accessible through Mendeley Data Repository [1]. The rationale behind offering these varied versions is to cater for a diverse audience of potential data users.

The first version, labeled ``MTE Tech Dataset_ Numeric.csv'' is provided in a .csv file format, facilitating easy access for a range of data analysis tools including Microsoft Excel. This dataset primarily contains numeric data for all variables. However, it is important to note that numeric data might not comprehensively represent the characteristics of specific variables, particularly in the case of categorical and ordinal variables. To address this limitation, the second version of the dataset, known as the ``MTE Tech Dataset_Text.csv'' includes textual data for both ordinal and categorical variables. In this file, numeric representations are reserved exclusively for scale variables like years of experience and age range. The third version, ``MTE Tch Dataset _SPSS.sav'' offers an added dimension but comes with a caveat – it may not be accessible to all potential users, as it requires the installation of SPSS software on their computers. Nevertheless, this version provides two distinct views: the variable view, which contains detailed information about each variable's features, and the data view, which presents the actual data for all variables.

The data within these datasets were collected using a semi-structured questionnaire, which is also openly available on Mendeley Data Repository [1]. This questionnaire exists in two versions: online (MTE Survey - Online version.pdf) and hard copy (MTE Survey- Hard copy version.pdf), both of which are provided in widely accessible PDF format. The hard copy version was designed to cater for respondents facing challenges in accessing the online version, which could stem from various factors, such as limited internet access or insufficient technological proficiency. This need for an alternative format arose, in part, from the data collected by Mukuka and colleagues [5] during the COVID-19 pandemic that pointed to a lack of internet access and limited technological expertise as prevalent issues among selected mathematics teachers and learners in Zambia.

The questionnaire consists of four parts namely, demographic information (Q1 – Q7), MTEs’ familiarity with various digital technologies (Q8 – Q10), MTEs’ proficiency with technology-based instruction (Q11 – Q14), and a section on perceived usefulness, perceived ease of use, and willingness (intention) to use technology (Q15 – Q18). Although this article primarily focuses on aspects other than demographic information, it is essential to acknowledge the significance of understanding the characteristics of the sample comprising the data providers. Additionally, it is important to highlight that, in compliance with the confidentiality commitments made to the respondents during the data collection phase, the actual names of the affiliated institutions have been redacted from the dataset.

Table 1 provides a breakdown of the respondents’ distribution across various demographic variables. All 104 respondents provided their gender and age range. However, one respondent omitted information about institutional ownership, while two did not specify the type of institution with which they were affiliated. Furthermore, nine respondents did not indicate their highest academic qualification.

**Table 1:** Respondents' demographic characteristics.

Variable	Frequency	Percent
Gender		
Male	82	78.8
Female	22	21.2
Total	104	100
Age		
Below 30	1	1.0
30–39	43	41.3
40–49	49	47.1
50–59	10	9.6
60 and above	1	1.0
Total	104	100
Institutional type		
College	51	53.7
University	44	46.3
Total	95	100
Institutional ownership		
Public	70	68.0
Private	33	32.0
Total	103	100
Highest qualification attained		
Bachelor	23	22.5
Masters	73	71.6
Doctorate	6	5.9
Total	102	100

The data presented in Table 1 reveals that a substantial majority of respondents were male, accounting for more than three-quarters of the total sample. Moreover, Table 1 indicates that over 88% of respondents fell within the age range of 30 to 49 years, with only 10.6% aged 50 and above, while a mere 1% were below 30 years old. This suggests that most of the surveyed Mathematics Teacher Educators are in the mid-career stage, likely with substantial teaching and educational experience. In contrast, a smaller proportion are aged 50 and above, indicating a smaller presence of older mathematics teacher educators.

Furthermore, the breakdown of the sample indicates that 51 respondents, comprising 53.7% of the total, were affiliated with colleges of education, while an additional 44 respondents (46.3%) were associated with universities.

In terms of qualifications, the majority of respondents, specifically 73 individuals (71.6%), held master's degrees, indicating a significant prevalence of MTEs with advanced postgraduate qualifications. Conversely, 23 respondents (22.5%) possessed bachelor's degrees, and a smaller group of 6 respondents (5.9%) held doctorate degrees, suggesting a relatively lower presence of MTEs with the highest academic credentials.

With respect to institutional ownership, a considerable majority of 70 respondents (68%) were affiliated with public institutions, while 33 respondents (32%) were part of privately owned institutions. This suggests a dominance of respondents teaching in publicly funded educational settings, with a smaller portion associated with private institutions.

The years of teaching experience among respondents ranged from 1 to 37 years (*M* = 9.5, *SD* = 6.8). Nevertheless, two exceptional cases, each with teaching tenures of 33 and 37 years respectively, were identified as outliers. Most respondents fell within the 1 to 25 years teaching experience range. This non-uniform distribution displayed a rightward skew, indicating a dominance of respondents with fewer years of experience, and was further underscored by a median teaching experience of 7 years.

## 4 Experimental Design, Materials and Methods

### 4.1 Formulation and validation of the survey items

As mentioned earlier, the questionnaire was structured into four distinct sections: demographic information (Table 1), familiarity with various digital technologies, proficiency in employing technology for mathematics instruction, and perceptions towards the utility, simplicity, and willingness to embrace technology in the classroom. Nonetheless, the emphasis of this paper is placed on the last two sections of the questionnaire. More specifically, this article delves into the MTEs' technology proficiency, their perceptions of technology's usefulness, ease of use, and willingness to incorporate technology into their teaching practices.

Before adapting the questionnaire items from existing research, the author recognized the potential variability in the interpretation of the term ``technology'' depending on the context. Consequently, this study narrowed its focus to digital technology, which is interchangeably referred to as ``technology'' or ``digital technology(ies)'' within this article. In line with Freiman's [6] perspective, digital technology is defined here as a combination of physically present 'hardware' devices (such as tablets, computers, printers, smartphones, projectors, etc.) and the associated 'software' or applications that facilitate user interaction with this hardware. According to Sinclair and Robutti [7], digital technology plays a dual role in mathematics education. Firstly, it aids in streamlining teachers' administrative tasks (e.g., generating worksheets, printing tests, and managing students' grades). Secondly, it fosters innovative approaches for creating and delivering mathematical content. In the context of this study, particular emphasis is placed on the second role that technology plays in the teaching and learning of mathematics.

The evaluation of proficiency in technology-based mathematics instruction encompassed four of the seven components from the TPACK model: technological knowledge (TK), technological content knowledge (TCK), technological pedagogical knowledge (TPK), and technological pedagogical content knowledge (TPCK) [3,^8^,9]. MTEs were requested to indicate their level of agreement with these components on a five-point Likert scale, ranging from 1 (strongly disagree) to 5 (strongly agree). In the final section of the questionnaire, MTEs were asked to indicate their level of agreement concerning the perceived usefulness (PU), perceived ease of use (PE), and their willingness or intention to incorporate technology (IT) into their teaching methodologies [2,10].

Before administering the questionnaire to the intended participants, it underwent quality assessment by five experienced researchers well-versed in the domain of technology integration in mathematics teaching and learning. These experts comprised two PhD holders and three PhD students, all of whom were contacted via email. The quality check process followed guidelines established in previously published works, with a particular emphasis on the “sufficiency, relevance, clarity, and coherence” of the questionnaire items [5,11].

The validators agreed that the initial questionnaire adequately (sufficiency) and pertinently (relevance) addressed the topics of educators’ proficiency, perceived ease of use, perceived usefulness, and willingness to use technology in the classroom. However, they expressed concerns about the clarity and coherence of some items, particularly those that were too generic and lacked a specific focus on mathematics teaching and learning. Another significant feedback was the questionnaire's omission to specify the technologies under consideration.

Upon receiving feedback from all five validators, their suggestions and comments were analyzed, integrated, and utilized to finalize the questionnaire [1]. The revisions included correcting spelling errors, rephrasing some statements to align with the questionnaire's focus, removing irrelevant items, and preparing both hard copy and online versions of the refined questionnaire. These changes not only enhanced the clarity and specificity of the questionnaire but also strengthened its alignment with the study's objectives.

### 4.2 Research design and setting

This study utilized a descriptive cross-sectional research design to collect data from Mathematics Teacher Educators (MTEs) in both private and public higher education institutions throughout Zambia. This design was aptly chosen as the study aimed to ascertain the present state of MTEs’ knowledge, attitudes, and practices concerning the integration of technology in mathematics teaching and learning. Furthermore, this design facilitated the accumulation of data that could serve as a benchmark for future research or interventions striving to enhance mathematics teacher training in Zambia.

The study specifically targeted lecturers who prepare teachers to teach mathematics at junior and senior secondary levels. Graduates of a college diploma program are equipped to teach mathematics to 8th and 9th graders, while those with a university bachelor's degree can teach mathematics to students in grades 8 through 12. According to the 2021 guidelines issued by the Higher Education Authority (HEA) [12], academic staff must possess a minimum academic qualification that exceeds the level of the learning program they are responsible for instructing. In practical terms, this implies that for an individual to be eligible to teach at a college of education in Zambia, they must hold a minimum of a bachelor's degree. This requirement aligns with the fact that colleges of education are tasked with the role of training diploma holders. Conversely, when it comes to teaching at the bachelor's degree level (mainly at a university), a master's degree is the minimum academic qualification. Therefore, the presence of bachelors, masters, and doctorate degree holders among the MTEs who participated in this study is justified, as it complies with the specified academic standards.

### 4.3 Research participants and data collection procedures

As mentioned earlier, the researcher developed a semi-structured questionnaire, grounded in the existing literature, and utilized Google Forms for its creation and distribution. The study adopted snowball and convenience sampling methods to recruit participants. While the author acknowledged the incongruity between the quantitative research approach and these sampling methods, it was anticipated that random sampling methods might not have provided an opportunity to maximize the sample size owing to the fact that the population of MTEs is quite small. Moreover, participation was voluntary. With the aim of recruiting as many participants as possible, employing random sampling was impractical. The researcher reached out to readily accessible MTEs, requesting them to disseminate the questionnaire link within their departments and other institutions. Assistance was also provided by some officials from the Ministry of Education to reach a broader audience. The data collection period spanned from mid-June to early-August 2023.

Data was obtained from 104 MTEs across 12 universities and 16 colleges of education in all ten provinces of Zambia. While this sample size adheres to the ten-times rule for structural equation modeling analysis [13], the researcher acknowledges that this rule may not be entirely reliable as it overlooks population size and other power analysis procedures [14,15]. Nonetheless, this sample is deemed adequate as it is representative of the target population for four key reasons: (1) the number of MTEs in recognized teacher training institutions is relatively small compared to educators in other subjects. (2) the sample encompasses higher learning institutions from all ten provinces of Zambia. (3) participation was voluntary, with only consenting individuals completing the questionnaire. (4) this sample size aligns with the specifications of the power analysis inverse method [14,15], based on path coefficients derived from similar previous studies [16]. This suggests that it is highly unlikely that a different sample from this population would yield drastically different results.

### 4.4 Model assessment criteria

The primary objective of collecting the data discussed in this paper was to establish the significance of the relationships among the four fundamental study constructs: proficiency, perceived usefulness, perceived ease of use, and willingness or intention to use technology. These relationships were strongly influenced by the Technology Acceptance Model (TAM), a highly influential theory in the realm of technology adoption, as noted by Davis [2].

According to the TAM, an individual's willingness to adopt technology is primarily determined by their perception of its usefulness and ease of use. However, recent research has shed light on external factors that can also impact these perceptions, as pointed out by Joo and colleagues [16]. Regarding the data described in this paper, the proficiency of MTEs with digital technology was considered an external variable that could influence how individuals perceive the usefulness and ease of use of technology, ultimately affecting their willingness to use it. These relationships are illustrated in Fig. 1, which depicts the hypothesized structural relationships.

Table 2 provides a breakdown of the indicators associated with each of the variables mentioned in Fig. 1, alongside their respective loadings. Proficiency, for instance, is represented by 16 indicators, while both perceived usefulness and perceived ease of use are characterized by six indicators each. Willingness or intention to use technology is indicated by three specific measures. The detailed statements corresponding to these indicators can be found in the hard copy version of the questionnaire provided on Mendeley Data [1].

**Table 2:** Factor loading from the initial model assessment.

Variable	Indicators	Factor loadings
Willingness	IT1	0.849
	IT2	0.827
	IT3	0.815
Perceived Ease of Use	PE1	0.841
	PE2	0.721
	PE3	0.851
	PE4	0.870
	PE5	0.807
	PE6	0.808
Perceived Usefulness	PU1	0.267
	PU2	0.892
	PU3	0.588
	PU4	0.657
	PU5	0.867
	PU6	0.540
Proficiency	TCK1	0.660
	TCK2	0.708
	TCK3	0.258
	TCK4	0.756
	TK1	0.805
	TK2	0.830
	TK3	0.679
	TK4	0.895
	TPCK1	0.841
	TPCK2	0.303
	TPCK3	0.715
	TPCK4	0.795
	TPK1	0.795
	TPK2	0.707
	TPK3	0.400
	TPK4	0.673

To ensure the reliability and validity of the model, an examination of the four variables presented in Fig. 1, whose respective indicators are detailed in Table 2 was carried out. This evaluation was conducted with the aim of offering valuable insights for other researchers looking to replicate this model in similar contexts, facilitating comparisons and the identification of commonalities and differences. The procedure for model assessment adhered to the guidelines outlined by Hair and colleagues [17] for evaluating the reliability and validity of the reflective measurement model. The model depicted in Fig. 1 was assessed using a consistent partial least squares structural equation modeling approach, resulting in the factor loadings displayed in Table 2. This approach ensures the robustness and accuracy of this analysis.

In the context of running a consistent Partial Least Squares Structural Equation Modelling (PLS-SEM) analysis, it is imperative to adhere to the recommended guideline that only indicators with factor loadings exceeding 0.708 should be retained, as suggested by Dijkstra and Henseler [18]. Based on the results displayed in Table 2, it is evident that all items pertaining to the variables ``willingness'' and ``perceived ease of use'' meet this criterion and thus should be retained. However, the variable ``perceived usefulness'' would retain only two out of its original six indicators if we strictly apply these guidelines. Similarly, the variable ``proficiency'' would retain just 10 out of its initial 16 items. This is why it is important to acknowledge the intricacies encountered in the social and behavioural sciences, as studies often involve respondents with diverse perspectives, attitudes, and varying levels of experience and competence. In light of this, it becomes particularly challenging to attain the ideal condition of retaining only indicators that meet the 0.708 factor loading threshold. Consequently, a more flexible approach of excluding only those indicators whose loadings posed a potential risk to the model's validity and reliability was adopted. For this reason, indicators with loadings below 0.5 were excluded from the subsequent model assessment.

Referring to the results in Table 2, only three indicators related to technological proficiency and one indicator associated with the perceived usefulness of technology were excluded from the second round of model assessment. These exclusions were necessitated by the compromise of the Average Extracted Variance (AVE) for these two variables, which were below 0.5, specifically yielding values of 0.448 and 0.491, as indicated in the supplementary file named ``Initial model assessment output'' This tailored approach ensures that while striving for model robustness, the unique complexities in the dataset are taken into account.

**Fig. 2:**
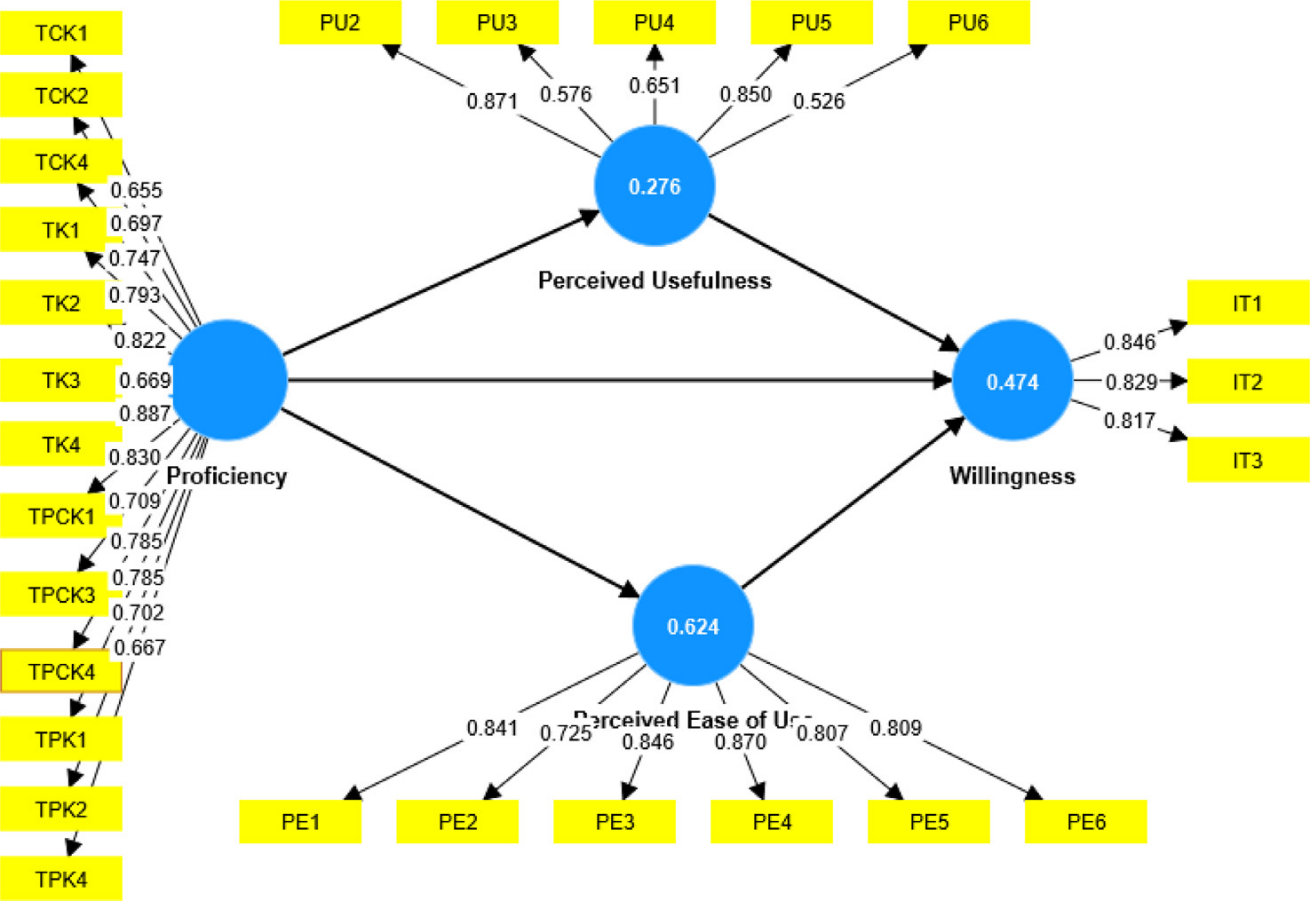
Factor loadings and coefficients of determination for the final model assessment.

Following the second run of the model, during which four indicators were excluded, it was observed that not all retained indicators displayed factor loadings exceeding the 0.708 threshold, as depicted in Fig. 2. Despite the apparent deviation from the strict factor loading guideline [4], there was no cause for concern as the model's validity was maintained. In other words, this deviation was offset by a significant improvement in the overall quality of the model. This improvement was manifested through the fulfillment of crucial criteria related to construct reliability and validity, discriminant validity, collinearity, and R square, as illustrated in Fig. 2, Table 3 and Table 4, along with the accompanying textual explanations. Further details about the fulfillment of the model's quality criteria are given in the supplementary file named “Final model assessment output”.

**Table 3:** Construct reliability and validity.

Variable	Cronbach's alpha	Composite reliability	Average variance extracted (AVE)
Perceived Ease of Use	0.923	0.924	0.669
Perceived Usefulness	0.812	0.829	0.503
Proficiency	0.944	0.944	0.567
Willingness	0.870	0.870	0.690

**Table 4:** Discriminant validity - Fornell-Larcker criterion.

Variable	Perceived Ease of Use	Perceived Usefulness	Proficiency	Willingness
Perceived Ease of Use	0.818			
Perceived Usefulness	0.649	0.709		
Proficiency	0.790	0.525	0.753	
Willingness	0.622	0.579	0.608	0.831

Besides displaying the factor loadings associated with each indicator, Fig. 2, provides insight into the Coefficients of Determination (R-square), which gauge the extent to which the explanatory variables can explain the total variation observed in the outcome variable. According to the guidelines by Hair and colleagues [19], an R-square value of 0.75 denotes a robust model fit, 0.50 signifies a moderate model fit, and 0.25 suggests a weaker model fit. However, it is worth noting that the interpretation of R-square values can be context-dependent, varying based on the specific discipline and research environment. To illustrate this contextual variance, a study like the one conducted by Raithel and colleagues [20] found an R-square value of 0.1 to be sufficient under certain circumstances. In the context of this model assessment, Fig. 2 reveals R-square values of 0.276 for the perceived usefulness of technology, 0.474 for the willingness to use digital technology, and 0.624 for the perceived ease of using digital technology. These values significantly exceed the recommended thresholds, indicating a robust model fit. This underscores the quality and appropriateness of the model for the specific context of the study.

Table 3 highlights that all constructs yielded composite reliability values between 0.70 and 0.95. These values, as established benchmarks, confirm the reliability of each construct in the model. Likewise, the Average Variance Extracted (AVE) values, that are normally used to assess convergent validity for each construct, were uniformly above the recommended threshold of 0.5 (Table 3). Convergent validity measures the extent to which a construct effectively converges to account for the variance of its constituent elements [17].

In terms of discriminant validity, the model exhibited good performance. The standard criterion for a robust model, as outlined by Fornell and Larcker [21], is that the square root of the AVE for each construct should surpass its correlations with other constructs. This condition was successfully met in this analysis, as demonstrated in Table 4. This achievement underscores the model's capacity to distinguish between different constructs and reinforces its overall validity and quality.

The supplementary file, titled ``Final model assessment output'' reveals that the Variance Inflation Factors (VIFs) ranged from 1.839 to 4.991. Notably, all VIFs calculated for each indicator remained below the threshold of 5. As per the guideline provided by Hair and colleagues [17], this signifies the absence of multicollinearity between any pair of indicators within each variable. In other words, the data does not suggest any problematic high correlations that could distort the reliability of the model, thus reinforcing the robustness of the model.

## Limitations

While the sample size for this study may not have been exceptionally large, it is essential to consider that participation in the research was entirely voluntary. This voluntariness factor is significant because many potential respondents, particularly MTEs, who are typically swamped with various responsibilities, may have chosen to decline the data collection request. Besides that, the target population size did not warrant a very large number of respondents.

Another noteworthy aspect is that the data used in this paper is based on self-reported responses from MTEs. Consequently, it is strongly advisable for future studies to complement this limitation by employing alternative data collection methods.

Amidst these two limitations, it is worth noting that the sample from which the data was drawn is representative of the target population especially that all the 10 provinces of Zambia were represented. Furthermore, the responses obtained in this study hold value for a wide spectrum of researchers, teacher educators, institutions involved in teacher training, and data analysts across diverse settings. As such, the data remains a valuable resource for a broad audience.

## Ethics Statement

This is to confirm that the relevant informed consent was obtained from the respondents. During the distribution of questionnaire, respondents were explicitly informed of the voluntary nature of their participation. They were also made aware that the data provided would be exclusively used for academic purposes, specifically for the dissemination of findings through publication in scholarly journals. Furthermore, prior to initiating the data collection process, ethical approval from the Walter Sisulu University Research Ethics Committee was granted. The ethical approval bears the protocol number **FEDSECC027-06-23** and is accessible in the supplementary files section, which accompanies this submission.

## CRediT Author Statement

**Angel Mukuka**: Conceptualization, Methodology, Data collection, Writing, Original draft preparation, and Writing- Reviewing. During manuscript preparation, generative artificial intelligence tools, specifically **ChatGPT 3.5** and **Bing Chat Enterprise**, were exclusively employed for language editing purposes. After using these tools, the author reviewed and edited the content as needed and takes full responsibility for the content of the publication.

## Data Availability

Mathematics Teacher Educators’ Technology Proficiency and Perceptions (Original data) (Mendeley Data).

## Appendix Supplementary materials

Image, application 1

Image, application 2

Image, application 3
