# Supplementary material for: Data on mathematics teacher educators’ proficiency and willingness to use technology: A structural equation modelling analysis
Source: Data Brief. 2024 Mar 11;54:110307. doi: 10.1016/j.dib.2024.110307 (PMC10957375; doi:10.1016/j.dib.2024.110307)
Supplement: Supplementary file 1 [file mmc1.pdf]

Directorate of Research Development and Innovation, Nelson Mandela Drive, Private Bag X1, MTHATHA 5117, South Africa. Tel: Tel: +27 47 502 2045. Email: research@wsu.ac.za

ETHICAL CLEARANCE CERTIFICATE

[ISSUED BY AUTHORITY OF UNIVERSITY RESEARCH ETHICS COMMITTEE, UREC]

**Protocol Number**	FEDSECC027-06-23
**Date of UREC Authorisation**	14 June 2023
**Certificate Date**	20/06/2023
**Valid until**	20/06/2023-20/06/2025<br>*(3 years from Certificate Date)*
**Title of Research Project**	Mathematics Teacher Educators' Expertise with Technology-based Instruction in Initial Teacher Training Context: Evidence from Zambia.
**Nature of Project** *(e.g. Doctor of Philosophy, Master of Social Work, Honours, Undergraduate research, Non-degree research)*	Non-degree research
**Principal Researcher**	Angel Mukuka
**Supervisor** *(where applicable)*	N/A
**Co-Supervisor** *(where applicable)*	N/A

Approval is hereby given by the Walter Sisulu University Research Ethics Committee (UREC) in respect of the ethical undertakings contained in the above-mentioned research project.

The Researcher(s) may therefore commence with the research as from the date of issue of this Certificate, using the above Protocol Number. Certificate validity is as indicated.

Please note that UREC must be notified immediately, should there be any material changes in the conditions or undertakings (such as research title, research site, research participants, research techniques, or data collection instruments).

Please quote the Protocol Number in all enquiries.

Dr T Saziwa (FRHDC CHAIR)

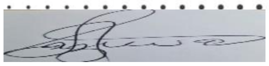

--- -----------------------------

Name & Designation of Authorised Signatory **Signature & Official Stamp**
